# Is There a Role for Bismuth in Diarrhea Management?

**DOI:** 10.5041/RMMJ.10422

**Published:** 2021-01-19

**Authors:** Helen Senderovich, Megan Vierhout

**Affiliations:** 1 Geriatrics & Pain Medicine & Palliative Care Physician, Baycrest Health Sciences, Toronto, Ontario, Canada; 2 Assistant Professor at the Department of Family and Community Medicine, Division of Palliative Care, University of Toronto, Toronto, Ontario, Canada; 3 McMaster University, Faculty of Health Sciences, Hamilton, Ontario, Canada

**Keywords:** Bismuth, diarrhea, inflammatory bowel diseases, quality of life

## Abstract

Diarrhea, an illness of both the developed and developing world, involves the burdensome characteristics of frequent bowel movements, loose stools, and abdominal discomfort. Diarrhea is a long-standing challenge in palliative care and can have a myriad of causes, making symptomatic treatment pertinent when illness evaluation is ongoing, when there is no definitive treatment approach, or when effective treatment cannot be attained. Symptomatic therapy is a common approach in palliative care settings. Bismuth is a suitable agent for symptomatic therapy and can be effectively employed for management of chronic diarrhea. The objective of this narrative review is to examine the role of bismuth in management of diarrheal symptoms. To explore this, PubMed (including Medline) and Embase were used to search the existing literature on bismuth and diarrhea published from 1980 to 2019. It was found that bismuth has potential utility for diarrheal relief in multiple settings, including microscopic colitis, traveler’s diarrhea, gastrointestinal infection, cancer, and chemotherapy. It also has great potential for use in palliative care patients, due to its minimal side effects. Overall, the antisecretory, anti-inflammatory, and antibacterial properties of bismuth make it a suitable therapy for symptomatic treatment of diarrhea. The limited range of adverse side effects makes it an appealing option for patients with numerous comorbidities. Healthcare providers can explore bismuth as an adjunct therapy for diarrhea management in an array of conditions, especially in the palliative care setting.

## INTRODUCTION

Diarrhea is an ailment associated with the symptoms of frequent bowel movements, loose stools, and abdominal discomfort.^1^ This illness affects both the developed and developing world.^2^ Due to the spectrum of its causes, especially in the palliative care (PC) setting, symptomatic therapy is a sensible approach when illness evaluation is still ongoing, when the diagnosis is lacking clear-cut treatment, or when effective treatment cannot otherwise be attained.^3^ Heavily centered on symptom relief, symptomatic therapy may be used in multiple settings of patient illness, especially PC. Chronic diarrhea has the potential to lower the quality of life of sufferers,^3^ which is of significant importance in PC, hence warranting effective management of its symptoms. Bismuth has the potential to alleviate chronic diarrhea and can be an effective symptomatic treatment in the PC setting.

There is a wide array of diarrheal causes, including viral, bacterial, and parasitic infections, irritable bowel syndrome, cancer, chemotherapy, and as a side effect of antibiotic therapy.^4^ Diarrhea is also known to be a main symptom of microscopic colitis (MC),^5^–^7^ which is a chronic inflammatory disease of the colon with a prevalence of 21–24.7 cases per 100,000 person years in the population.^8^ This disease is becoming a more prevalent focus for gastroenterologists, and there has been an enhanced rate of detection.^5^ Bismuth has been observed to have positive outcomes on diarrhea caused by MC.^5^–^7^ Bismuth has also been a longstanding therapy for the treatment of gastrointestinal symptoms.^9^ As a derivative of salicylic acid, bismuth subsalicylate (BSS) has antibacterial properties,^7^,^10^ making it suitable and effective as a treatment for MC-induced and other forms of diarrhea. However, it is not available in all countries due to risks of long-term toxicity.

In PC settings, symptomatic therapy is an emphasized priority. Diarrhea is especially dangerous in this setting, as terminally ill patients are already compromised and are at increased risk for malnutrition, dehydration, electrolyte loss, and fatigue.^11^,^12^ Various factors may contribute to diarrhea in PC, especially side effects of treatment for the primary illness.^13^,^14^ Symptom and pain relief are ranked high in the top indicators for improved quality of life in PC.^15^ Based on the literature, some of bismuth’s applications relate to the management of diarrhea of different etiologies (including traveler’s diarrhea, gastrointestinal infection, MC, irritable bowel syndrome, surgery, and non-specific diarrhea). However, it can be used in cancer and chemotherapy as a symptomatic therapy, indicating a beneficial role of bismuth in PC, which merits further exploration.

Overall, there is limited previous literature on the use of bismuth in diarrhea management, especially in the field of PC. Our paper offers novelty in the field as it explores diarrhea management with bismuth in multiple settings, including applications of bismuth as a symptomatic therapy in PC. This is applicable to clinical issues related to diarrhea and may offer insight into management options when a first-line option has been ineffective or contraindicated.

This narrative review therefore examines the role of bismuth in management of diarrheal symptoms in various settings, with a focus on MC-induced diarrhea. The properties of bismuth with regard to its advantages in the management of multifactorial diarrhea will be discussed in light of the literature.

## METHODS

**Figure 1:**
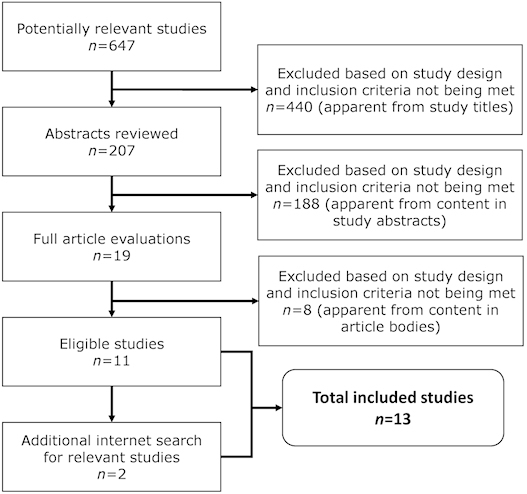
Breakdown of Study Selection. A detailed breakdown of the numbers for potentially relevant studies, abstracts reviewed, full article evaluations, and eligible studies obtained from PubMed and Embase literature searches.

Searches were performed on PubMed (including Medline) and Embase (Elsevier) for recent studies on “bismuth” OR “bismuth subsalicylate” AND “diarrhea,” between January 1980 and September 2019. All searches were also limited to “English language,” “human,” and “clinical trial.” This yielded over 600 results, including reviews, case reports, letters to the editor, book chapters, animal studies, clinical trials, and randomized control trials. The search was further narrowed (Figure 1), and specific inclusion/exclusion criteria (Table 1) were applied. Additional papers were located from the references of papers and additional general Internet searches using Google, Google Scholar, and Caresearch, and the above strategies. Additional searches were performed for specific topics, such as diarrhea and PC, to locate topics not directly related to bismuth and diarrhea.

**Table 1:** Inclusion and Exclusion Criteria for Reviewed Studies.

Inclusion Criteria	Exclusion Criteria
English language	Language other than English
Studies involving human subjects	Animal studies
Primary studies	Non-primary articles
Studies involving adults (≥18 y)	Studies involving children and participants under 18 years of age
Studies with statistically significant findings (*P*<0.05)	Studies without statistically significant findings
Studies including development of diarrhea, diarrheal incidence, or stool frequency as an outcome	Studies focused on *Helicobacter pylori*

All studies were reviewed by two independent reviewers to determine their eligibility. Only primary studies conducted on human subjects with observed parameters directly related to diarrhea were included. These included prospective, retrospective, randomized double-blind (RDB), and placebo-controlled studies. Review articles and commentaries were not included. In all studies, bismuth was a main form of intervention being trialed. Eligible studies were read and summarized, and the study design, population, parameters observed, and outcomes documented (Table 2).

**Table 2:** Relevant Studies Involving Bismuth in Diarrhea Management.

Author (Year), Reference	Study Design	Population	Parameters Observed	Conclusions
Fine & Lee (1998)^5^	Prospective cohort	13 MC patients (7 with subepithelial collagen deposition and 6 without)	Stool, histological analysis, blood tests	Eight-week BSS treatment is an effective intervention for MC
Gentile et al. (2015)^16^	Retrospective	64 MC patients	Response rate based on baseline diarrhea severity	MC patients treated with BSS had a strong response rate, but they were also at high risk to have recurrence
Steffen et al. (1988)^17^	RDB, parallel study	245 tourists with traveler’s diarrhea	Stool timing, stool consistency, number of stools, relief of therapy timing	BSS was well tolerated and effective in shortening the duration of traveler’s diarrhea
Steffen et al. (1986)^18^	RDB study	390 travelers (aged 16–70 y) going to a developing country	Incidence of diarrhea, microbiological results	Incidence of diarrhea in the treatment groups was significantly lower than in the placebo group, and bacteria was only found in the stools of the placebo group
DuPont et al. (1987)^19^	Randomized, placebo-controlled trial	182 students from USA in Mexico	Development of diarrheal illness, occurrence of mild illness, protection rate, pathogen in stool	Overall, BSS was an effective and safe treatment to reduce the occurrence of traveler’s diarrhea
DuPont et al. (1980)^20^	RDB placebo-controlled study	150 students traveling to Mexico	Occurrence of diarrhea, mild changes in stool form, enteropathogen detection	There was a protective effect of BSS on occurrence of diarrhea and infection with enteropathogens
Johnson et al. (1986)^21^	Randomized trial	296 adult students diagnosed with acute diarrhea	Number of unformed stools by hour, response to therapy, symptomatic relief	For treatment of traveler’s diarrhea, loperamide is an effective alternative to BSS; the median number of unformed stools was less in subjects taking loperamide compared to BSS
Graham et al. (1983)^22^	RDB placebo-controlled study	32 healthy volunteers inoculated with enterotoxigenic *E. coli*	Development of diarrhea, symptomatic response, stool weight, stool frequency, antibody titer	BSS can be effective in prevention of traveler’s diarrhea
Hansen & Penkowa (2017)^23^	RDB, prospective pilot study	50 hematological inpatients	Duration of diarrhea and gender-linked incidence	In lymphoma patients, bismuth significantly reduces diarrhea relative to placebo
Zaveri et al. (2018)^24^	Prospective RDB, placebo-controlled, crossover study	36 patients with flatus and/or stool odor changes who were at least 6 months post-loop duodenal switch	Gastrointestinal Quality of Life Index (GIQLI) questionnaire	For the treatment group GIQLI overall scores and digestive scores were significantly higher after treatment; quality of life after the treatment with bismuth was significantly higher compared to pre-treatment and post-placebo
Daghaghzadeh, et al. (2018)^25^	Placebo-controlled clinical trial	129 IBS patients with three subtypes: IBS-constipation dominant (IBS-C), IBS-diarrhea dominant (IBS-D), and IBS-mixed (IBS-M)	IBS-related questionnaires, IBS-severity scoring system	Quality of life significantly improved in IBS-D from study beginning to end; bismuth had a significant effect on improvement of IBS-D patient symptoms
Hernández et al. (2015)^26^	Prospective, observational study	65 patients undergoing biliopancreatic diversion with Scopinaro’s classic technique	Gastrointestinal Quality of Life Index (GIQLI) questionnaire, nutritional status	All domains of the GIQLI were significantly higher after bismuth treatment than before treatment; in patients undergoing Scopinaro’s biliopancreatic diversion, bismuth subgallate was effective for short-term treatment of unfavorable symptoms
DuPont et al. (1990)^27^	Open-label, parallel comparison study	203 adult students from USA or Mexico diagnosed as having acute, non-specific diarrhea	Average number of unformed stools, stool frequency, time to last unformed stool (TTLUS), time to first unformed stool (TTFUS), percent of participants with no additional loose stools and requiring no additional doses	Loperamide is more effective for acute non-specific diarrhea relief than BSS; mean TTLUS was lower for loperamide and median TTFUS was longer; loperamide patients overall required fewer doses compared to BSS patients

BSS, bismuth subsalicylate; IBS, irritable bowel syndrome; MC, microscopic colitis; RDB, randomized double-blind.

### Case Vignettes

Three demonstrative case vignettes were also developed to illustrate potential applications of bismuth in clinical settings. The vignettes presented below involve multifactorial origin of diarrhea, where treatment with bismuth could be attempted. We then aligned the vignettes with the findings of this review, supported by references.

#### Vignette 1

This patient was a male in his 60s with a long-standing history of MC. He was a resident of long-term care (LTC) and was bed-bound. He presented with loose bowel movements (BM), five to six times/day, in addition to other comorbidities such as anxiety and irritable bowel syndrome. His medications included sulfasalazine, budesonide, mirtazapine, gabapentin, and ferrous fumarate.

The patient utilized loperamide for a long time prior to admission to LTC, with some response. Following LTC, resins such as cholestyramine were attempted with partial relief of symptoms, reducing BM frequency to three or four times daily, but requiring a large volume of fluids with each cholestyramine dose. This led to poor cholestyramine adherence, and BM were only partially controlled. He could not tolerate budesonide and developed syncope. Bismuth was introduced at an initial dosage of 262 mg orally once per day, and then titrated up to 524 mg four times a day (2,096 mg total daily). Bowel movement frequency decreased to once or occasionally twice daily, and the patient stabilized. His mood, as well as quality of life, significantly improved. A bout of aspiration pneumonia and antibiotic treatment led to relapse of diarrhea while being off bismuth; bismuth was resumed for another month. No exacerbation of MC was noted during that time. The bismuth was titrated down during the next three months to 120 mg orally twice daily, and despite the reduction his BM remained under control. Alternation of bismuth intake continued for the patient, with a one-month break in bismuth treatment, following by three months of bismuth, 120 mg orally twice daily; no recurrence of diarrhea was noted, resulting in improved quality of life.

#### Vignette 2

This patient was a female in her 90s residing in the PC unit of a LTC facility. She had a history of loose BM five to six times daily and multiple comorbidities including lymphoma. Her medications included ferrous fumarate, duloxetine, and hydromorphone. Mood management was challenging as mood fluctuated on a daily basis and was closely related to her BM pattern. Loose BM and abdominal discomfort restricted her ability to participate and attend recreational activities that she had previously enjoyed. In addition, she was experiencing repeated falls secondary to orthostasis, and possible dehydration induced by the high frequency of diarrhea. Her care goals were comfort and symptom control with a priority for quality of remaining life.

Bismuth was introduced at 1,000 mg twice a day for five days orally; the patient soon stabilized without any negative sequelae. Her mood improved, and she was able to preserve her quality of life. Bismuth was titrated down then to 500 mg twice a day for the next 10 days, and she remained stable. No worsening symptoms were noted. Treatment was stopped when oral intake was lost and she approached end of life.

#### Vignette 3

This patient was a female in her 80s being cared for in a PC unit of a LTC facility. She had a history of recto-sigmoid cancer with severe diarrhea—14 to 16 BM daily. She had undergone radiation therapy complicated by radiation proctitis two years prior to admission. Unfortunately, the tumor continued to progress. Her medications included ferrous fumarate, proton pump inhibitors, hydromorphone, and tranexamic acid. The patient’s behavior shifted daily with restlessness and agitation frequently observed, which appeared to be related to BM frequency. The discomfort associated with her diarrhea restricted her ability to participate in events that brought joy and added value to her life. In addition, she was experiencing frequent melena, and persistent severe uncontrollable diarrhea that was escalating the intensity of rectal bleeding. Patient 3 became increasingly fatigued, was emotionally depressed due to the lack of improvement in her condition, and her quality of life was significantly reduced.

Bismuth 1,000 mg twice a day for five days was attempted, and she stabilized soon after. However, the patient also developed tremor, visual hallucinations, severe nausea, blackening of the tongue and stool, and began to decline rapidly cognitively. Bismuth was reduced to 500 mg twice a day, with only partial symptom improvement: tremor and visual hallucinations ceased, but blackening of the tongue and stool persisted and severe nausea continued to negatively impact quality of life. Bismuth was discontinued, thereby resolving the nausea and blackening of the tongue and stool, but there was a recurrence of her diarrhea.

Charcoal, 262 mg orally once daily, was initiated, in addition to octreotide 900 mg daily divided in three doses. Frequency and consistency of BM improved; soon after the octreotide was discontinued, while charcoal was titrated up to an oral amount of six doses daily. Bowel movement frequency decreased to four to five times daily, and its consistency changed from loose to soft. With the diarrhea no longer disturbing her, the patient’s mood and behavior improved, as well as the quality of her life.

## RESULTS

Thirteen studies were found to be relevant to the objective at hand; these are listed in Table 2. Eleven of these studies were located utilizing PubMed and Embase, and two were found with additional general Internet searches. These studies focused on the utilization of bismuth in various settings, including two studies on MC,^5^,^16^ five on traveler’s diarrhea,^17^–^21^ and one each on gastrointestinal infection,^22^ chemotherapy and cancer,^23^ loop duodenal switch,^24^ irritable bowel syndrome,^25^ Scopinaro’s biliopancreatic diversion,^26^ and acute, non-specific diarrhea.^27^ The majority of studies found bismuth to be beneficial in diarrhea management. Two studies compared loperamide and bismuth and found loperamide to be more effective for acute diarrhea and traveler’s diarrhea.^21^,^27^

## DISCUSSION

### Mechanism of Action

The exact antidiarrheal mechanism of action has not yet been determined for bismuth. However, BSS is thought to have antidiarrheal action through antisecretory, antibacterial, and anti-inflammatory operations, as it is a derivative of salicylic acid.^7^,^28^ Bismuth has been seen to promote fluid and electrolyte absorption across the intestinal tract,^28^ and significantly inhibit fluid accumulation in the intestinal lumen.^29^ By reducing the amount of free fluid, diarrheal symptoms are ameliorated. Bismuth is also described to have adsorbent properties.^29^ A study observing the effects of bismuth on the various bacterial pathogens of *Clostridium difficile*, *Salmonella*, *Shigella*, and Shiga toxin-producing *Escherichia coli* showed reduced bacterial growth *in vitro* after treatment with bismuth, compared to untreated control.^30^ Bismuth has also been observed to adhere to the toxins synthesized by *E. coli*.^28^

### Microscopic Colitis

As mentioned above, MC is an inflammatory condition of the bowel, which has had an increasing incidence^31^,^32^ and a growing rate of detection by gastroenterologists.^5^ There are two MC subtypes: collagenous colitis, where a thickened subepithelial collagen band is present, and lymphocytic colitis, where it is absent.^5^,^7^,^8^,^31^–^33^ The exact pathogenesis is unknown^5^,^7^,^32^,^34^; however, pathogenic and commensal bacteria^5^ as well as autoimmune components are believed to play a role in MC.^5^,^8^,^31^,^32^

### Microscopic Colitis and Diarrhea

Microscopic colitis is a considerably prevalent source of chronic diarrhea,^5^–^8^,^16^,^31^,^33^ and unfortunately this symptom can be extremely wearying to patients. The mechanism of diarrhea in MC is speculated to be attributed to multiple elements, including the severity of the inflammatory response, impairment of electrolyte absorption, and increased secretion.^32^ Additionally, increased levels of prostaglandins in the lumen and mucosa may also be associated with inflammatory diarrhea caused by MC.^32^

In addition to being known to promote fluid and electrolyte absorption in the intestinal tract,^28^ bismuth is able to inhibit prostaglandin synthesis,^28^ and it has both anti-inflammatory and antibacterial properties. For all these reasons, bismuth seems to be an appropriate candidate for treating MC. However, there remains a knowledge gap regarding MC therapy,^5^ especially lymphocytic colitis.^33^ Commonly used MC drugs include sulfasalazine and mesalamine; however, one of the associated side effects for these drugs is diarrhea itself.^5^ Budesonide is the most studied drug for MC^31^ and is a corticosteroid therapy to which the majority of patients respond.^6^–^8^ Although it is a first-line therapy, some studies have shown adverse effects,^34^ as seen in the case of Vignette 1 where the patient developed syncope, and patients taking budesonide long-term should be monitored. In a study completed by Fine and Lee,^5^ bismuth not only reduced the frequency and weight of bowel movements, but also improved stool consistency, and worked at a histopathological level to reduce tissue abnormalities and inflammation. The resolution of inflammation at the histological level by bismuth was also seen in a case study involving collagenous colitis.^35^ Gentile et al.^16^ studied both collagenous and lymphocytic colitis patients who were treated with bismuth. They found decreased diarrhea severity that was dose-related: higher doses (up to nine tablets daily) were more effective than lower ones, which was also observed in all demonstrative cases (Vignette 1, Vignette 2, and Vignette 3). In all of the MC and bismuth studies reviewed, bismuth reduced diarrhea in MC patients,^5^,^16^,^33^,^35^ which was also seen in the case of Vignette 1.

### Traveler’s Diarrhea and Gastrointestinal Infection

Traveler’s diarrhea is the most prevalent illness related to travel and is caused by bacterial, viral, or protozoal pathogens.^36^ Approximately 80% of traveler’s diarrhea cases are attributed to bacterial infection,^10^,^37^ with enterotoxigenic and enteroaggregative *E. coli* being the most common bacterial pathogens involved in all areas except Southeast Asia.^36^ Common viral pathogens include norovirus and rotavirus.^36^ Bismuth can be taken for chemoprevention^10^,^36^,^37^ and can prevent up to 65% of cases of expected traveler’s diarrhea.^10^,^36^ It can also be taken after the onset of traveler’s diarrhea and has been shown to reduce diarrhea duration.^17^ In other studies, bismuth prevented the growth of pathogens related to traveler’s diarrhea in adults visiting other countries.^18^,^19^ In another RDB, placebo-controlled study, diarrhea and the presence of enteropathogens in the stool were reduced in subjects taking bismuth.^20^ It is believed that the bactericidal mechanisms of bismuth may involve cell wall degradation, inhibition of plasma membrane function, and prevention of adenosine triphosphate (ATP) synthesis.^30^ These antimicrobial properties suggest the suitability of bismuth for the treatment and prevention of traveler’s diarrhea.^22^ However, it should also be noted that a study examining the effects of probiotic *Saccharomyces boulardii* and bismuth on cholera revealed that neither of the therapies, nor their combination, was suggested as an adjunct treatment for cholera, due to the study being underpowered and lacking statistical significance between study arms.^38^ These pathogens are also of concern for infections in the PC and LTC settings.

### Cancer and Chemotherapy

Cancer-associated diarrhea can be attributed to a multitude of treatment sources, including chemotherapy, signal transduction inhibitors, immunotherapy, radiotherapy, laxatives, surgery, and infection.^39^,^40^ Diarrhea can also be a symptom of the cancer itself, such as in adrenal,^41^ pancreatic,^42^ lung neuroendocrine, and colorectal cancers.^39^ Cancer is a common ailment in PC, and many patients may experience diarrhea as a symptom of their illness. This was seen in the case of the patient in Vignette 3, who experienced severe diarrhea due to her recto-sigmoid cancer. This symptom may actually interfere with the cancer treatment itself.^40^ Since bismuth promotes the absorption of fluid^28^ and inhibits fluid accumulation in the intestines,^29^ it is an appropriate proposed therapy for cancer and chemotherapy-induced diarrhea where there is an excess of fluids present. In a RDB, prospective pilot study by Hansen and Penkowa, the effects of bismuth on patients receiving cancer treatment revealed that the duration of diarrhea experienced by lymphoma patients receiving melphalan chemotherapy was decreased as compared to the placebo group.^23^ However, in patients with multiple myeloma, diarrhea persisted irrespective of treatment.^23^ This suggests that the effects of bismuth on cancer-related diarrhea may be contingent on the type of cancer, and that lymphoma patients specifically can benefit from bismuth’s antidiarrheal effects, as was seen in the case of the patient in Vignette 2 who suffered from lymphoma.

### Side Effects

Pardi et al. reported that non-specific antidiarrheal agents including bismuth had limited side effects.^33^ Several studies reported that bismuth therapy is safe and well-tolerated by patients^5^,^20^,^23^,^34^; of note is the prospective cohort study of Fine and Lee, confirming this.^5^ In PC settings, patients often have multiple complications and symptoms, therefore treatment-related side effects should be avoided. This was seen in Vingette 1, where the patient developed the side effect of syncope with budesonide, but was able to tolerate bismuth well. However, caution should be exercised with chronic use of this medication, especially at high doses, as rare but serious side effects related to neurotoxicity have been reported. These include impaired cognitive function, tremors, myoclonus, visual hallucinations, and gait impairment.^43^ Other reports of toxicity include delirium, psychosis, ataxia, and seizures.^44^ This was seen in the patient of Vignette 3, who experienced cognitive impairment, tremor, and visual hallucinations with the use of bismuth, which required her to discontinue bismuth and begin to take charcoal for diarrhea management. Although bismuth toxicity is very infrequent, awareness should still be promoted due to its severity and the availability of the drug on the market. Furthermore, bismuth should not be given to patients with renal impairment, who may experience symptoms of neurotoxicity, neuromuscular spasm, neuromuscular weakness, hearing loss, and gastrointestinal issues induced by bismuth accumulation.^45^ Due to the known risk of long-term toxicity, bismuth is unavailable in many countries,^31^ with bismuth subgallate use being restricted in France and banned in Australia.^46^ Additionally, the very ability of bismuth to promote fluid absorption in the intestinal tract could also precipitate constipation in some cases, such as when it is administered as a preventative.^18^ Other non-adverse side effects that have been observed are objectionable taste,^18^ nausea,^18^ and blackening of the tongue and stool.^10^,^36^,^37^

### Limitation of Use

Although bismuth appears to be an effective drug for the management of diarrhea, it is not without limitations. For traveler’s diarrhea prevention, it must be taken frequently (two to four times per day)^36^ and in a large quantity (eight tablets),^47^ making it a less appealing choice for travelers. It has also been known to compromise the absorption of other medications, including doxycycline for example.^47^ Side effects, especially for healthy individuals taking the medication as a preventative measure, such as blackened tongue and tinnitus, also make bismuth use less desirable.^47^ Long-term therapy with bismuth raises concerns for adverse side effects and toxicity, including cognitive and gait impairment, tremors, myoclonus, visual hallucinations, delirium, psychosis, ataxia, and seizures.^43^,^44^ Bismuth cannot be used in patients with renal impairment.^45^ Lastly, studies have also found that loperamide is more effective than bismuth for the management of acute diarrhea^26^ and showed more symptom relief in traveler’s diarrhea.^21^ However, bismuth showed effectiveness for traveler’s diarrhea in multiple studies,^17^–^20^ and, unlike loperamide, its safety with comorbid common *C. difficile* infections in PC settings should be appreciated.

## CONCLUSIONS

In conclusion, this review points to bismuth having suitable potential as a symptomatic therapy for diarrhea. Diarrhea can be a symptom or side effect of many causes, including cancer, chemotherapy, antibiotics, gastrointestinal infections, irritable bowel syndrome, traveler’s diarrhea, and MC. Bismuth’s anti-inflammatory and antibacterial properties make it a useful therapy in these settings. Additionally, since diarrhea in palliative settings can be multifactorial with many confounding factors, symptomatic therapy, such as through treatment with bismuth, can be a logical approach. Minimal side effects have been associated with bismuth therapy, and serious reactions are rare, making it a suitable option for PC patients experiencing diarrhea. However, chronic use at high doses should be monitored as neurotoxic side effects are a possibility. It should also be noted that bismuth treatment should be used with caution in PC, where patients may have impaired renal function. Overall, the safety and tolerability of bismuth can be appreciated in PC, in the setting of a fragile population with multiple comorbidities and complications. However, it should also be recognized that restriction of bismuth use in some parts of the world is a roadblock for a potentially useful treatment of PC patients in these countries. Further research is required in order to evaluate the efficacy of bismuth in palliative settings, and overall as a therapy for diarrheal illness.

### Lessons Learned

Due to its antisecretory, anti-inflammatory, and antibacterial characteristics, bismuth is equipped with the properties to manage diarrhea from multiple angles. Its overall limited range of adverse side effects makes it an appealing option for patients with numerous comorbidities. Healthcare providers can explore bismuth as an adjunct therapy for diarrhea management in an array of conditions. Interestingly, patients with older age at the time of MC diagnosis are more likely to have a positive outcome when using bismuth than are younger patients,^48^ which also supports the use of bismuth in elderly palliative populations. In infectious diarrhea, bismuth can be added to antibiotic treatment due to its antimicrobial properties.^11^ *Clostridium difficile* and *E. coli* infection, in both of which bismuth can be utilized, frequently occur at LTC facilities and hospices.^49^,^50^ Interestingly enough, in a study conducted on bacterial infection in hospice patients, *E. coli* was found to be the most frequent pathogen.^49^ Bismuth offers a viable option as a first-line treatment instead of loperamide, which cannot be used in the case of *C. difficile* infections, common in PC, as it may cause megacolon and escalate the severity of colitis.

### Further Rese**a**rch

Further studies involving clinical evaluation are critical to determining the efficacy and potency of bismuth in the aforementioned settings and conditions. Firstly, it would be beneficial to obtain a clearer understanding of the mechanism of action of bismuth, as its exact antidiarrheal mechanism has not been determined. Basic science research should be conducted to elucidate the antidiarrheal mechanism of bismuth, which may also provide further insight and understanding of the appropriate situations for its use.

Although the described studies involve various types of diarrhea, including that caused by MC, traveler’s diarrhea, bacterial infection, chemotherapy, duodenal switch, irritable bowel syndrome, and Scopinaro’s biliopancreatic diversion, no studies have evaluated the effectiveness of bismuth in palliative settings. It is important to explore the use of bismuth as a symptomatic therapy, such as through randomized control trials, to determine optimal dosing and usage as well as the possible challenges. Further research should also be conducted to better define the barriers associated with bismuth. For example, there is a need to further explore the application of bismuth in patients with renal impairment. Studies are required to investigate the safety of this symptomatic therapy in palliative patients.

Further research is also required to determine the specific types of cancers and associated chemotherapeutics where bismuth may be suitable to manage cancer and chemotherapy-related diarrhea. Additional randomized control and placebo-controlled studies would be beneficial to determine against which other types of bacterial and viral infections bismuth may be effective. This is also applicable to PC, where overall more randomized control trials with larger sample sizes and longer follow-up times are needed to determine the role of bismuth in this setting.

## Abbreviations

ATP: adenosine triphosphate
BM: bowel movements
BSS: bismuth subsalicylate
*C. difficile*: *Clostridium difficile*
*E. coli*: *Escherichia coli*
LTC: long-term care
MC: microscopic colitis
PC: palliative care
RDB: randomized double-blind
